# MPP7 mediates EMT via Wnt/β-catenin pathway to promote polarity changes in epithelial ovarian cancer cells

**DOI:** 10.7150/jca.96185

**Published:** 2024-06-17

**Authors:** Chunlin Tao, Xiaoge Ni

**Affiliations:** 1 Department of Obstetrics and Gynecology, Shanghai Jiao Tong University Affiliated Sixth People's Hospital South Campus, Shanghai, China.; 2 Department of Obstetrics and Gynecology, Affiliated People's Hospital of Jiangsu University, Zhenjiang, China.

**Keywords:** Epithelial Ovarian Cancer, MPP7, Wnt/β-catenin, EMT, Cell Polarity

## Abstract

Ovarian cancer is one of the gynecological malignancies with the highest mortality rate. Its widespread metastasis is difficult to cure, and the beneficiaries of targeted therapy are still limited, which has been a long-standing bottleneck problem. MAGUK P55 scaffold protein 7 (MPP7) plays an important role in the establishment of epithelial cell polarity, but its potential significance in epithelial ovarian cancer is still unclear. In this study, we investigated the expression profile of MPP7 and its functional role in epithelial ovarian cancer. Through analysis of TCGA and GEO databases, combined with immunohistochemical staining of ovarian tumor tissue chips, it was found that MPP7 is significantly overexpressed in epithelial ovarian cancer tissue, and its high expression is closely related to poor prognosis of patients. It has been verified through cell function experiments that interference with MPP7 can inhibit the proliferation, migration, and invasion of ovarian cancer cells *in vitro*. Performing planar polarity immunofluorescence staining on ovarian cancer cells revealed that interference with MPP7 can cause polarity changes in ovarian cancer cells. The transcriptome sequencing results of the ovarian cancer database were analyzed, and Western Blot was used to verify that MPP7 may mediate EMT via Wnt/β-catenin signaling pathway and promote changes in cell polarity in human epithelial ovarian cancer, thereby promoting cancer progression, demonstrating the potential of MPP7 as a new biomarker and target for the diagnosis and treatment of ovarian cancer.

## Introduction

Ovarian cancer is one of the most malignant gynecological tumors, and it is expected that new cases of ovarian cancer in the United States will account for 2% of all malignant tumors in women with a mortality rate of 4% in 2024 1. From 2000 to 2018, although the overall cancer mortality rate in China decreased by an average of 1.3% per year, the annual average percentage change (AAPC) of ovarian cancer was 4.4%, still showing an upward trend 2. Epithelial ovarian cancer (EOC) is the most common type of ovarian cancer, accounting for about 90% of the total number of ovarian cancers. According to the histological types of epithelial ovarian cancer, it can be divided into serous, endometrioid, mucinous and clear cell carcinoma 3. One of the main reasons for the high mortality rate of ovarian cancer is its susceptibility to metastasis 4, 5. During the process of solid tumor cell metastasis, different types of polarities are established, including apical basal polarity and anterior posterior polarity 6, 7. Cell polarity refers to the asymmetry of morphology and molecules 8, providing a structural basis for cell adhesion and communication 9. Studies have shown that the occurrence and metastasis of tumors are closely related to cell polarity, and the absence of epithelial cell polarity contributes to the occurrence of tumors 10-12. Changes in the expression patterns of polarity related proteins have been shown to affect epithelial polarity, thereby regulating cell proliferation, migration, and tumor development 13.

Membrane associated guanosine kinase (MAGUKs) proteins are a class of scaffold related proteins that exist in the cell-cell contact region and play a role in cell adhesion, tight junctions, and polarity 13, therefore, it is closely related to tissue development and various physiological processes 14. The MAGUK protein family can be divided into several subfamilies based on its structural characteristics, including the membrane palmitoyl protein (MPP) subfamily 15.

The Crumbs (CRB) - Stardust (SDT) - Discs lost (DLT) complex of Drosophila plays a crucial role in the establishment and maintenance of epithelial polarity, and MPP1-MPP7 in the membrane palmitoyl protein (MPP) subfamily is a human homolog of Drosophila SDT 16. SDT is a type of membrane associated guanosine kinase (MAGUK) protein with L27, PDZ, SH3, and GuKc domains. DLT is an adapter protein with MAGUK recruitment (MRE) domains and multiple PDZ domains. SDT binds to DLT through the interaction of L27 and MRE domains, promoting the formation of epithelial cell polarity and tight junctions 17, 18.

Recent studies have shown that MPPs also play a role in tumor development, for example, MPP2 can regulate the activity of the oncogene SSRC 19. Downregulation of MPP6 can improve anti-cancer activity caused by Saa3 gene knockout 20, while MPP6 can inhibit the progression of ovarian cancer simultaneously 21. MPP7 can promote the progress of pancreatic cancer by regulating autophagy 22, and can activate epidermal growth factor receptor (EGFR) / AKT (also called protein kinase B, PKB) signal to promote the progress of breast cancer 23. Therefore, we have developed a keen interest in the role of the MPPs subfamily in ovarian cancer. This study aims to preliminarily explore the main members of the MPPs subfamily that promote the progression of epithelial ovarian cancer and explore their mechanisms of action in ovarian cancer.

## Material and methods

### Bioinformatics analysis

This study conducted differential gene analysis between ovarian tumor tissue and normal ovarian tissue using 'The Cancer Genome Atlas' (TCGA) combined with the 'Genotype Tissue Expression' (GTEx) database to screen for target genes. Then we further analyzed the expression of the target gene in pan cancer using R language to clarify its statistical significance of abnormally high expression in ovarian cancer, and conducted in-depth analysis of the expression levels of target genes in normal ovarian tissue and ovarian cancer tissue using multiple datasets from the 'Gene Expression Omnibus' (GEO) comprehensive database. Finally, survival analysis was conducted using the Kaplan Meier plotter database to find out the impact of high expression of the target gene on progression free survival (PFS) and overall survival (OS) in ovarian cancer patients.

### Organizational chip immunohistochemistry

The ovarian tumor tissue samples used in this study were provided by patients who voluntarily joined the study, signed an informed consent form for the experiment, and included complete case information (142 patients with benign ovarian epithelial cysts and 96 patients with epithelial ovarian cancer, aged 16-83 years old female; we collected relevant pathological specimens from Shanghai Jiao Tong University Affiliated Sixth People's Hospital South Campus from 2012 to 2020). Inclusion criteria for patient enrolled: All cases of ovarian tumors were diagnosed by pathologic as well as epithelial origin. Exclusion criteria (conform to one of the following two conditions): ①Patients had received preoperative chemotherapy, radiotherapy, immunotherapy, etc. ②Patients or family members refused to provide pathological tissue samples and personal clinical information. All sample collection and experimental design had passed the qualification review of the Ethics Committee of Shanghai Jiao Tong University Affiliated Sixth People's Hospital South Campus. Suzhou Xinxin (Biotechnology) Co., Ltd. in Jiangsu Province was entrusted to produce all ovarian tumor tissue chips.

Staining method and specific process are as follows: We baked the tissue chips at 60 °C in advance overnight, cooled them down, and then placed them in xylene solution for dewaxing (10 minutes, 2 times). Then soaked them in a 1:1 mixture of xylene and anhydrous ethanol, anhydrous ethanol, 75% anhydrous ethanol, 50% anhydrous ethanol, and ultrapure water for 5 minutes each for hydration. We placed the chips in 0.01 M (pH 6.0) sodium citrate antigen repair solution and boiled them to 95-100 °C for 20 minutes for antigen repair. After natural cooling, we rinsed them twice with PBS, added 0.3% H_2_O_2_- methanol (mixed 3% hydrogen peroxide (MKBio, MM0750) and methanol solution in a 1:9 ratio) dropwise on the surface of the chips, and incubated them in dark for 10 minutes to eliminate endogenous peroxidase activity. We rinsed chips with PBS solution (5 minutes, 3 times), and added 2% BSA blocking buffer (MPBIO, 0218072801) dropwise onto them to block at room temperature for 1 hour. We incubated the chips overnight with a primary antibody at 4 °C. We rinsed the chips with PBS solution the next day (5 minutes, 3 times), and incubated them at room temperature for 1 hour after adding the secondary antibody dropwise. We rinsed with PBS solution (5 minutes, 3 times), prepared a colorimetric solution (A:B=1:1) using DAB horseradish peroxidase assay kit (Beyotime, P0203), added the colorimetric solution dropwise onto the chips and stained for 3-4 minutes. Once the chips' color rendering was satisfactory, they were quickly rinsed with flowing water to terminate the colorimetric process. We shook off excess water and stained chips with hematoxylin (Beyotime, C0105S) for 1-3 minutes. We then rinsed continuously with water for 5-10 minutes. We added 1% HCl - 75% ethanol mixture droplet onto the chips for about 15 seconds for differentiation, and then rinsed continuously with running water. We soaked the chips in ultrapure water, 50% anhydrous ethanol, 75% anhydrous ethanol, anhydrous ethanol, a 1:1 mixture of xylene and anhydrous ethanol, and xylene for dehydration treatment, and placed them in a fume hood to dry. We sealed and saved chips with neutral resin.

The immunohistochemical staining results were determined by two senior pathologists who read the slides and scored the positive intensity of the staining effect on each array point under a microscope (based on brown 3 points, brown yellow 2 points, light yellow 1 point, and no staining 0 points). Next, the coloring area of the chip was scored (based on the coloring area > 2/3, = 1/3 ~ 2/3, < 1/3, and no coloring, scoring 3 points, 2 points, 1 point, and 0 points in order). We added up the two scores and evaluated the staining results. If the total score was less than 3, it indicated low expression, and if the total score was greater than or equal to 3, it indicated high expression.

### Cell culture

The ovarian cancer cell lines were gifted by Professor Zhang Zhigang from the Cancer Institute of Shanghai Jiao Tong University. The cell lines were amplified and tested for Mycoplasma. The cells were passaged 2-3 times a week and cultured until the 20th generation. According to the different culture conditions of each cell line, they were cultured in RPMI 1640 (BasaMedia, L210KJ, containing 2.0g/L D-glucose and 300mg/L L-glutamine) or DMEM (BasaMedia, L110KJ, containing 4.5g/L D-glucose, 4 mM L-glutamine, and 0.11g/L sodium pyruvate) containing 10% fetal bovine serum (FBS) (BI, 04-001-1A04-001-1A). After screening, the human ovarian cancer cell lines used in this study were OVCAR8 and ES2. The cell culture incubator was set at 37 °C with a 5% CO_2_ concentration.

### SiRNA transient interference

We inoculated ovarian cancer cells into a 6-well plate with appropriate density, when transfected, it was advisable to achieve a confluence ratio of about 50%. We precipitated the siRNA freeze-dried powder to the bottom of the tube, and added 250μl of RNase free ddH2O according to the instructions of Ruibo Company to dissolve the siRNA oligonucleotides. We added 5 µl of Lipofectamine 3000 reagent (Invitrogen, L3000-008) to 250 µl Opti-MEM media (Gibco, 11058021) and let it stand at room temperature for 5 minutes. Then we added 5 µl si-RNA to 250 µl Opti-MEM media and let it stand at room temperature for 5 minutes. We mixed the above two premixes and let them stew at room temperature for 15 minutes. We removed the supernatant culture media from the 6-well plate, transferred 1ml of fresh complete media into each well, and added the above-mentioned transfection reagents separately. We shook the 6-well plate using the "Z" method and mixed well, then placed the plate in a cell culture incubator for further incubation. We changed the media 6-8 hours later, transfected for 48 hours, and extracted RNA and protein for PCR and western blot experiments to detect interference efficiency, or for subsequent experiments.

Negative control (si-NC) was scrambled siRNA, its sense sequences were 5'-UUCUCCGAACGUGUCACGUdTdT-3' and antisense sequences were 5'-GCGACGAUCUGCCUAAGAUdTdT-3'. The sequences of siRNA oligonucleotides for MPP7 were shown as follows: si-MPP7-1 sense sequences were 5'-GGATACCAGTGGAGGATAA-3' and antisense sequences were 5'-UUAUCCUCCACUGGUAUCC-3', si-MPP7-2 sense sequences were 5'-GCACAAGTATAGACTCAGT-3' and antisense sequences were 5'-ACUGAGUCUAUACUUGUGC-3', si-MPP7-3 sense sequences were 5'-GGAGCAATTACATTTAAGA-3' and antisense sequences were 5'-UCUUAAAUGUAAUUGCUCC-3'.

### Western blot

The cells were cultured until 90% confluence in the petri dishes, then we removed the culture media, and rinsed the cells 1-2 times with PBS solution pre cooled at 4 °C. After preparing protein lysis solution containing 10 mM 100× protease inhibitor/phosphatase inhibitor (Bimake, B15001) and RIPA buffer (Beyotime, P0013B) in proportion, we added the above solution to the petri dishes (200 µl in a 6cm petri dish and 500 µl in a 10cm petri dish). We let it lyse on ice for 10 minutes. We scraped off the cells with a scraper and transferred them to a 1.5ml eppendorf tube, then inserted it on ice to lyse for 30 minutes. We centrifuged the eppendorf tube at 4 °C and 13400 × g for 15 minutes. We transferred the supernatant liquid to a new eppendorf tube using a pipette, and performed sample addition detection using the BCA protein concentration detection kit (Beyotime, P0010) according to the instructions. We calculated the protein sample concentration by measuring the OD value combined with the protein standard curve using an enzyme-linked immunosorbent assay (with a set wavelength of A562nm). According to the amount of protein samples collected, we added 5 × protein loading buffer (Beyotime, P0015L) and boiled at 100 °C for 5-10 minutes. We let it cool at room temperature and stored it at -80 °C or -20 °C, or directly used for WB detection on the same day.

We prepared SDS-PAGE gel with PAGE gel rapid preparation kit (EpiZyme, 10%, PG112), added protein samples, and conducted protein electrophoresis according to the set voltage. The protein contained in OC cell lysis solution was divided with accurate SDS-PAGE gels. After transferring onto nitrocellulose (NC) membrane (PALL, 0.45u), the protein was blocked by 5% skim milk within TBS/Tween-20 (Tris Buffered Saline (TBS, Powder): Servicebio, G0001. Tween-20: Sigma, P7949) for 1 hour. After that, we washed the NC membrane with TBST three times (10 minutes each time). The NC membrane was further cultured with primary antibody such as anti-MPP7 (1:1000, Proteintech, 12983-1-AP), anti-Wnt10b (1:10000, Proteintech, 67210-1-Ig), anti-β-caternin (1:10000, Proteintech, 51067-2-AP), anti-P-β-catenin (Ser33) (1:1000, Proteintech, 28772-1-AP), anti-c-MYC (1:5000, Proteintech, 10828-1-AP), anti-N-cadherin (1:5000, Proteintech, 22018-1-AP), anti-E-cadherin (1:20000, Proteintech, 20874-1-AP), anti-snail1 (1:1000, Proteintech, 13099-1-AP), anti-Vimentin (1:5000, Proteintech, 10366-1-AP ) and anti-GAPDH (1:10000, Abcam, ab181602) overnight at 4 °C. The next day, we removed the NC membrane and washed it three times with TBST (10 minutes each time). Then added the corresponding secondary antibody and incubated it on a shaking table at room temperature for 1 hour. After washing, the NC membrane was treated with a 1:1 ECL luminescent solution (Share bio, LumiQ General, SB-WB012). We placed it in an imaging device for development. The grayscale values of the stripes were quantitatively analyzed using Image J software.

### Polymerase chain reaction (PCR)

We extracted RNA according to the instructions of the RNA rapid extraction kit (Share bio, SB-R001), and measured RNA concentration using NanoDrop. We took 500ng RNA according to the measured concentration and dissolved it in Primerscript^TM^ RT Master 5× Mix (Takara, RR036A) 2 μl and RNase Free water to form 10 μl reverse transcription system followed by RT-PCR. The obtained cDNA was mixed and diluted with RNase Free water in a 1:30 ratio. 4.2 μl Diluted cDNA, corresponding primers (0.4μl for each forward primer and reverse primer) and 2× SYBR Green qPCR Master Mix 5 μl (Bimake, B21402) were taken to configure to 10 μl system (per well in 96-well plate), with 3 repeated wells per sample, and were subjected to RT-qPCR according to the set reaction conditions. The Ct value of the amplification curve was determined by the cyclic logarithm ΔRn of PCR. We calculated the specific expression level of each relative gene using 2^-ΔΔCt^ (ΔCt = Ct value (target gene) -Ct value (RPS18)). Finally, t-test was used for statistical analysis, with P < 0.05 indicating significant differences (RT-PCR reaction conditions: 37 °C, 30 minutes for reverse transcription, 85 °C, 30 seconds for reverse transcriptase inactivation, 4 °C, unlimited time. RT-qPCR reaction conditions: stage 1: 95 °C, 5 minutes, one cycle. stage 2: 95 °C for 10 seconds, 60 °C for 60 seconds, 40 cycles. stage 3: 95 °C for 15 seconds, 60 °C for 1 minute, 95 °C for 15 seconds. Forward primer for RPS18 was ATCACCATTATGCAGAATCCACG, reverse primer for RPS18 was GACCTGGCTGTATTTTCCATCC, forward primer for MPP7 was AGAACCACTGGGAGCTACCAT, reverse primer for MPP7 was CCCGTTGACTTCCCTAAGTTCAT).

### Cell proliferation experiment

#### Cell Counting Kit-8 (CCK8) experiment

The ovarian cancer cells were seeded in a 96-well plate (2000 cells and 100 µl of complete media per well) and incubated overnight in a cell culture incubator. The next day, we removed the culture media from each well and replaced it with 100 µl of fresh complete media containing 10% CCK-8 reagent (Targetmol, C0005). We incubated in dark for 1-4 hours, after equilibrating the temperature of the microplate reader to 37 °C, the OD value was detected at 450 nm. We measured every 24 hours for 4-5 days. We drew a growth curve using GraphPad based on the measured OD value, and performed statistical analysis on the data.

#### Colony formation assay

1000 cells were seeded in a 6-well plate with 2ml of complete media per well. We blew cells apart to form a single-cell state, and incubated overnight until the cells were completely attached to the dish. The next day, we observed the cell density and colony formation, continue to culture for 1-2 weeks, and changed the media every 2-3 days. When a single ovarian cancer cell proliferated into a cell cluster and a clear clone cell cluster was visible to the naked eyes, we fixed cells with 4% paraformaldehyde (Biosharp, BL539A) for at least 30 minutes. After washing, we stained cells with 0.1% Crystal Violet Staining Solution (Solarbio, G1063) for at least 30 minutes. We rinsed repeatedly with running water to remove residual staining solution and dried the 6-well plate in the air. We observed and took photos under a microscope, counted using Image J software, and performed statistical analysis.

### Cell migration/invasion experiment

#### Cell migration experiment

We placed the transwell chamber (Corning, 353097) in a matching 24-well plate. 2-5×10^4^ cells were seeded in the upper chamber with 200 µl of complete media, while 700 µl of corresponding culture media (including 20-30% FBS) was put into the lower chamber. We incubated for 16-24 hours. We removed the culture media from the upper and lower chambers respectively, and soaked the chambers with PBS for 3 times. Then we fixed and stained cells, wiped off the cells in the upper chamber with a cotton swab, after drying, we observed the cells penetrating the chamber under a microscope, randomly took photos of six fields of view, counted them using Image J software, and performed statistical analysis.

#### Cell invasion experiment

We placed the matrix gel (Corning, 354234) at 4 °C and thawed it in advance. We took an appropriate amount of matrix gel and diluted it with pre cooled DMEM media without FBS in a ratio of 1:40. We placed 100 µl of diluted matrix glue on the inner side of each transwell chamber, and then put the 24-well plate into the incubator for 2 hours to allow the matrix glue to solidify into a membrane. All steps before putting into the incubator needed to be carried out on ice, and labwares needed to be pre cooled in advance. The remaining steps were the same as the cell migration experiment.

### Planar polarity immunofluorescence staining

#### Cell scratch

Cells were seeded in a 24-well plate with 1ml of complete media per well to incubate overnight. On the second day, when the cell confluence reached about 90%, we used a 200μl pipette tip combined with a ruler to make vertical scratches at the bottom of the dish. We washed twice with PBS to remove cells that had fallen off from scratches. We replaced with fresh complete media and continued incubating overnight.

#### Cellular immunofluorescence staining

After removing the culture media and washing, we fixed cells with 4% paraformaldehyde for at least 30 minutes. We penetrated with 0.1% Triton100 (Beyotime, P0096) for 10 minutes. We then configured primary antibody (GM130, 1:50, proteintech, 11308-1-AP) with 1% BSA, added 200μl to each well, incubated overnight at 4 °C. The next day, we added 200μl of fluorescent secondary antibody (APExBIO, K1209) to each well, and incubated at room temperature in dark for 1 hour. We stained the nucleus with DAPI (APExBIO, C3362) at a concentration of 1g/ml for 3-5 minutes. We added 1-2 drops of Antifade Mounting Medium (Beyotime, P0126) to each well, observed and took photos under an inverted fluorescence microscope.

### Statistical analysis

IBM SPSS Statistics 25 and Graph Pad Prism 9 version software (San Diego, CA) was used for statistical analyses. Data were expressed as the means ± SEM. Data comparisons between groups was performed by One-way ANOVA or two-tailed student's t-test. *P* < 0.05 were perceived as statistically significant.

## Results

### Expression of membrane palmitoyl protein (MPP) subfamily in ovarian cancer

**Figure 1:**
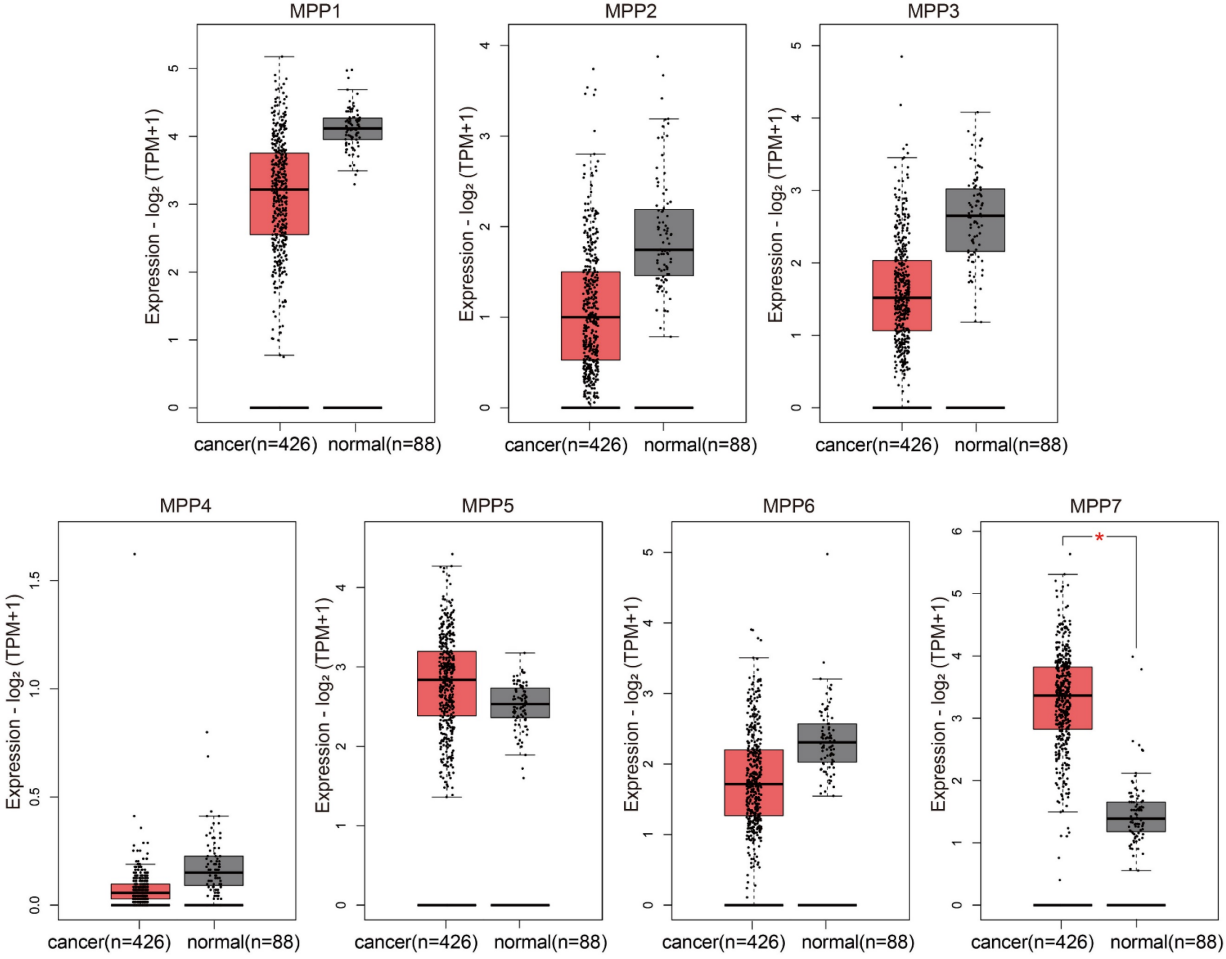
TCGA/GTEx database analysis of the expression of MPP1-MPP7 in ovarian cancer.

We used bioinformatics methods to analyze the expression of MPP1-MPP7 in TCGA ovarian cancer and GTEx normal ovaries databases, and found that only the expression level of MPP7 was significantly increased in ovarian cancer samples (Fig. 1).

Compared to normal ovarian tissue, only the expression level of MPP7 in MPP1-MPP7 was significantly increased in ovarian cancer samples (*P* < 0.01).

### MPP7 was highly expressed in epithelial ovarian cancer and was associated with poor prognosis in patients

**Figure 2:**
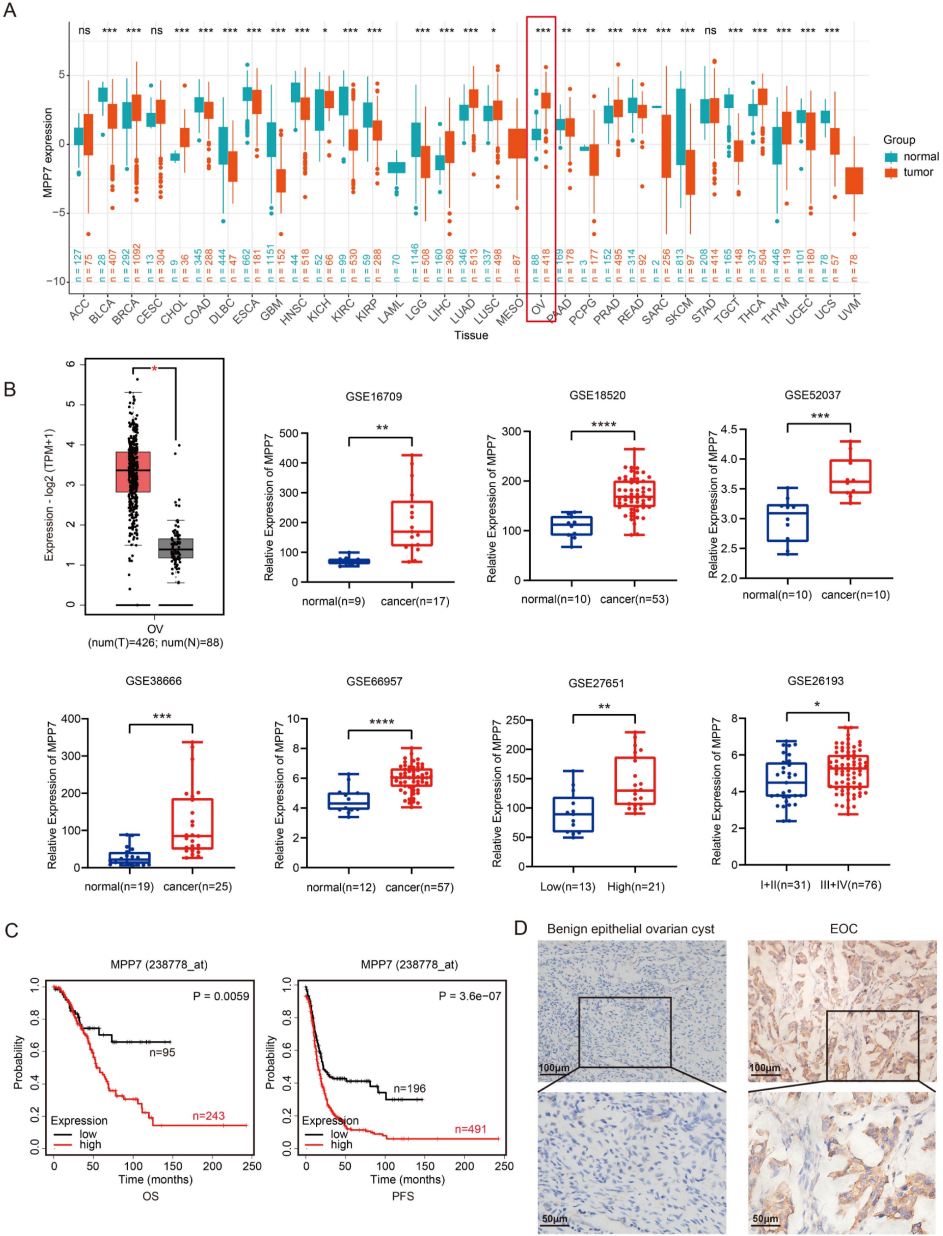
** Expression and prognostic analysis of MPP7 in epithelial ovarian cancer**. **A:** The expression of MPP7 in pan cancer; **B:** The expression of MPP7 in various datasets of TCGA/GTEx and GEO databases; **C:** Survival analysis of MPP7 using Kaplan Meier plotter database; **D:** Immunohistochemical staining of MPP7 in benign epithelial ovarian cysts and epithelial ovarian cancer tissues (*: *P* < 0.05, **: *P* < 0.01, ***: *P* < 0.001, ****: *P* < 0.0001)

By analyzing the expression levels of MPP1-MPP7 in pan cancer, we found that MPP7 was significantly overexpressed in ovarian cancer tissue (Fig. 2A, *P* < 0.001); Further analysis of the expression levels of MPP7 gene in 88 normal ovarian tissues in the GTEx database and 426 ovarian cancer tissues in the TCGA database revealed significant high expression of MPP7 in ovarian cancer tissues (Fig. 2B, *P* < 0.05). Further analysis was conducted on the expression levels of MPP7 in normal ovarian tissue and ovarian cancer tissue using the GSE16709, GSE18520, GSE52037, GSE38666, GSE66957, GSE27651, and GSE26193 datasets in the GEO database, the results also showed that compared with normal ovarian tissue, the expression level of MPP7 was significantly increased in ovarian malignant tumor tissue (Fig. 2B, *P* < 0.05~ *P* < 0.0001). Furthermore, through survival analysis using the Kaplan Meier plotter database, we found that compared with progression free survival (PFS) in patients with low MPP7 expression, ovarian cancer patients with high MPP7 expression had a poorer prognosis (Fig. 2C, *P* < 0.0001). Similarly, compared to the overall survival (OS) of patients with low MPP7 expression, ovarian cancer patients with high MPP7 expression also had a poorer prognosis (Fig. 2C, *P* = 0.0059). In order to investigate the clinical significance of MPP7 in ovarian cancer, we used tissue chip immunohistochemistry to detect the expression of MPP7 in tissue samples from 142 patients with benign ovarian epithelial cysts and 96 patients with ovarian epithelial cancer. The results showed that compared with ovarian cysts, MPP7 had a higher expression level in ovarian cancer samples (Fig. 2D). Among them, in benign epithelial ovarian cyst samples, the proportion of MPP7 high expression was only 7.7%, while in ovarian cancer samples, the proportion of MPP7 high expression was 63.5%, *P* < 0.0001, and the difference was statistically significant (Table 1). Next, we analyzed the relationship between the expression of MPP7 and the clinical and pathological characteristics of epithelial ovarian cancer. The results showed that compared with low-grade ovarian cancer, MPP7 had higher expression levels in high-grade ovarian cancer. In low-grade ovarian cancer, the proportion of MPP7 overexpression was 19.4%, while in high-grade ovarian cancer, the proportion of MPP7 overexpression was 84.6%, *P* < 0.0001 (Table 1).

Compared with early (Ⅰ+Ⅱ) ovarian cancer patients, MPP7 had higher expression levels in samples of late (Ⅲ+Ⅳ) ovarian cancer. Among early ovarian cancer samples, the proportion of high expression of MPP7 was 31.9%, while in late ovarian cancer samples, the proportion of high expression of MPP7 was 93.9%, *P* < 0.0001 (Table 1). Compared with cases without lymph node metastasis, MPP7 had a higher expression level in cases with lymph node metastasis. In cases with negative lymph nodes, the proportion of high expression of MPP7 was 38.2%, while in cases with positive lymph nodes, the proportion of high expression of MPP7 was 97.6%, *P* < 0.0001 (Table 1). In summary, MPP7 was significantly overexpressed in epithelial ovarian cancer tissue, and its high expression was closely related to late clinical stage, high pathological grade, and lymph node metastasis. MPP7 might be an effective indicator for predicting poor prognosis in ovarian cancer patients.

### The biological function of MPP7 in epithelial ovarian cancer

**Figure 3:**
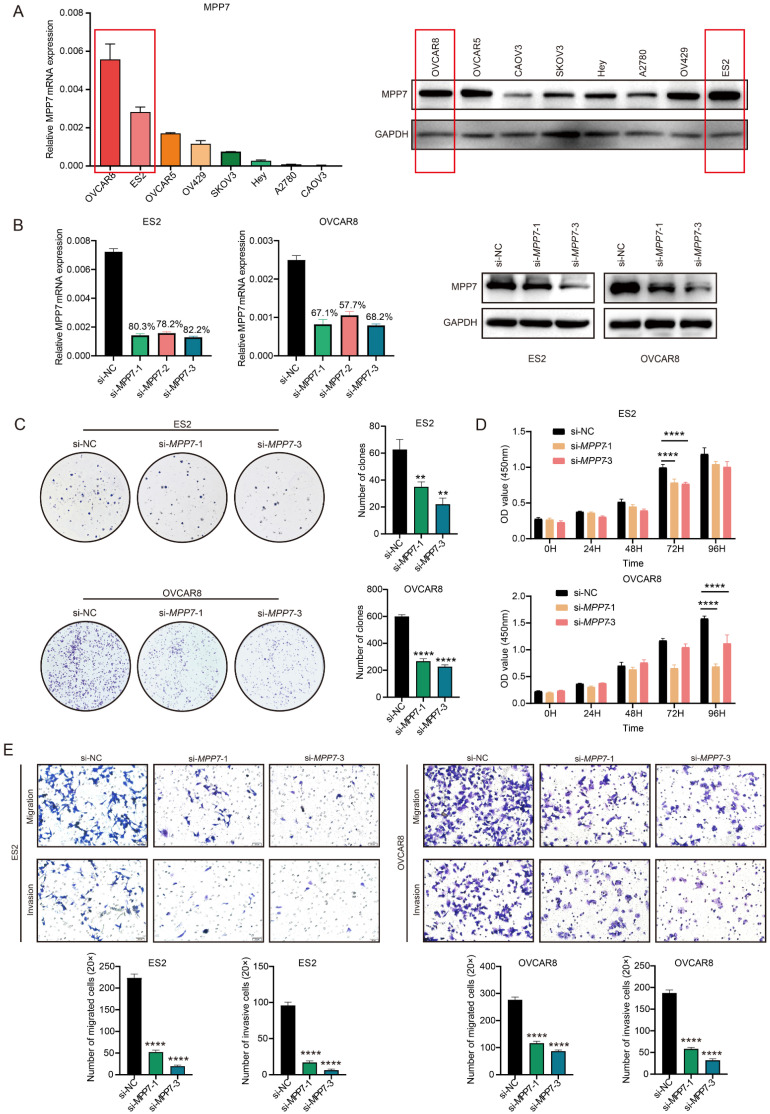
** Biological function of MPP7 in epithelial ovarian cancer**. **A:** Analysis of MPP7 expression at mRNA and protein levels in 8 types of epithelial ovarian cancer cells; **B:** Transient interference with MPP7 expression in OVCAR8 and ES2 cells, and verification of MPP7 expression at mRNA and protein levels respectively; **C:** Plate cloning experiments showed that interfering with the expression of MPP7 can significantly inhibit the proliferation of ovarian cancer cells *in vitro*; **D:** CCK8 assay showed that interfering with the expression of MPP7 could significantly inhibit the proliferation of ovarian cancer cells *in vitro*; **E:** Transwell cell migration/invasion experiments had shown that interference with MPP7 could significantly inhibit the migration and invasion ability of ovarian cancer cells *in vitro*. (*: *P* < 0.05, **: *P* < 0.01, ***: *P* < 0.001, ****: *P* < 0.0001)

We analyzed the expression of MPP7 in 8 types of epithelial ovarian cancer cells and found that MPP7 expression was higher in OVCAR8 and ES2 cells (both at mRNA and protein levels) (Fig. 3A). Therefore, we chose to perform transient interference on the expression of MPP7 in these two types of epithelial ovarian cancer cells, and then verified the biological function of MPP7 in ovarian cancer. Firstly, after interfering cells with three fragments of si-MPP7, the interference efficiency was verified at the mRNA and protein levels. It was found that si-MPP7-1 and si-MPP7-3 had better interference efficiency (Fig. 3B). Selected these two interfering fragments to knock down the expression of MPP7 in OVCAR8 and ES2, and conducted cell proliferation, migration, and invasion experiments. The results showed that the proliferation ability of ovarian cancer cells with low expression of MPP7 was significantly inhibited. In the plate cloning experiment, the number of cell proliferation was less than 50% of the control group (*P*_(ES2)_ < 0.01, *P*_(OVCAR8)_ < 0.0001, Fig. 3C). In the CCK8 experiment, the two interference groups of cells showed a significant decrease in proliferation ability at 72 hours and 96 hours respectively (*P* < 0.0001, Fig. 3D). In the cell migration / invasion experiments, we found that knocking down MPP7 significantly inhibited the migration and invasion ability of ovarian cancer cells by statistically analyzing the number of cells that penetrated each group (*P*_(ES2-Migration)_ < 0.0001, *P*_(ES2-Invasion)_ < 0.0001, *P*_(OVCAR8-Migration)_ < 0.0001, *P*_(OVCAR8- Invasion)_ < 0.0001, Fig. 3E). The above results indicated that interfering with the expression of MPP7 could inhibit the proliferation, migration and invasion of ovarian cancer cells *in vitro*.

### Molecular mechanism exploration of MPP7 promoting ovarian cancer progression

**Figure 4:**
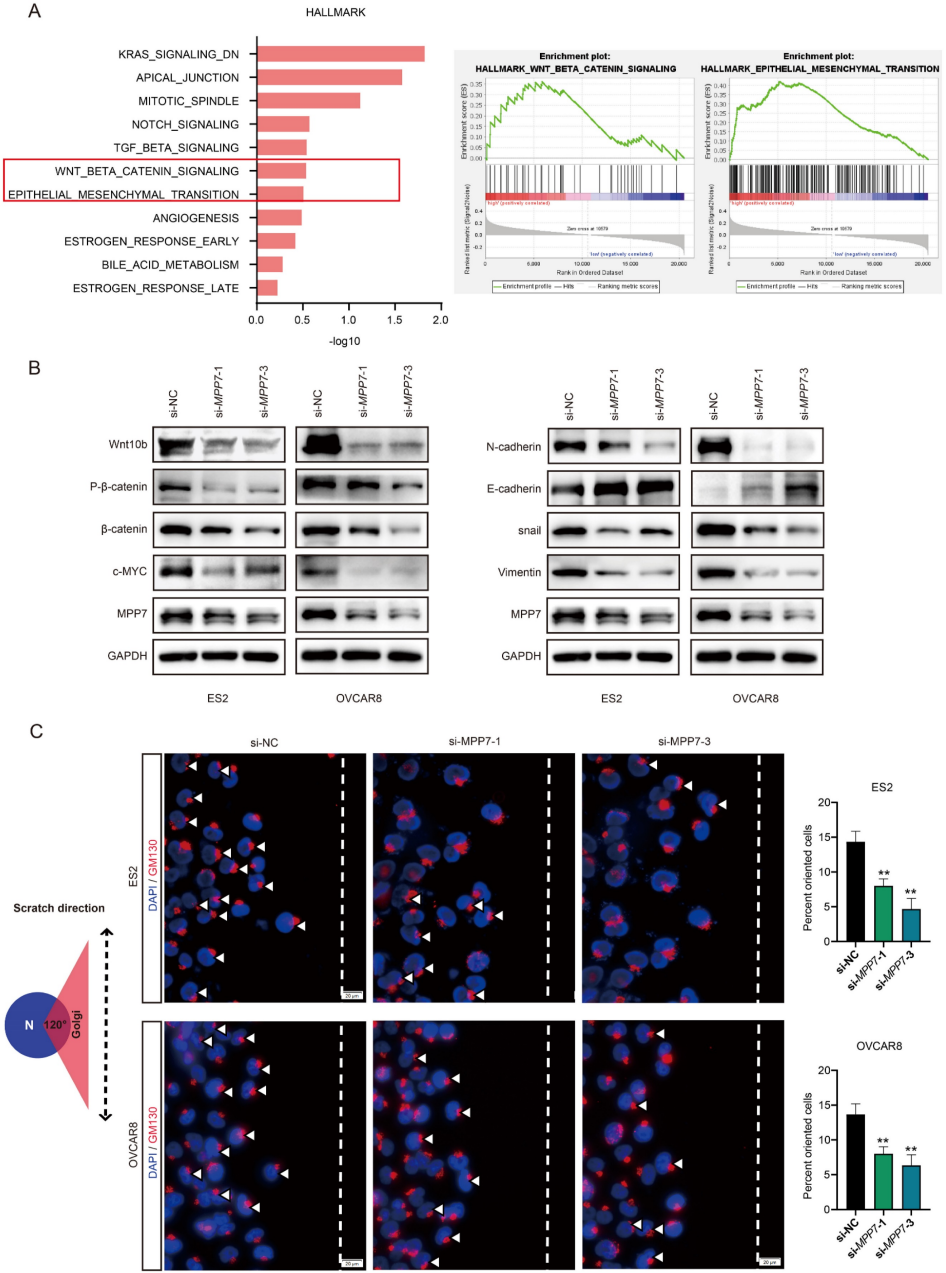
** Preliminary exploration of the molecular mechanism of MPP7 promoting ovarian cancer progression**. **A:** GESA analysis of RNA sequencing data in the TCGA ovarian cancer database; **B:** Instantaneously interfered with the MPP7 of epithelial ovarian cancer cells OVCAR8 and ES2, and verified the related pathway proteins of Wnt/β-catenin and EMT; **C:** Schematic diagram of cell directed migration, changes in polarity and cell directed migration rate of ovarian cancer cells after interference with MPP7 through immunofluorescence co-localization analysis. (*: *P* < 0.05, **: *P* < 0.01, ***: *P* < 0.001, ****: *P* < 0.0001)

We conducted GESA analysis on RNA sequencing data in the TCGA ovarian cancer database and found that MPP7 was associated with Wnt/β-catenin signaling pathway and epithelial mesenchymal transition (EMT) (Fig. 4A). Next, we instantaneously interfered with the MPP7 of epithelial ovarian cancer cells OVCAR8 and ES2, and verified the related pathway proteins of Wnt/β-catenin and EMT. The results showed that knocking down MPP7 resulted in the expression of Wnt10b, β-catenin and c-MYC being significantly reduced, while the expression of phosphorylated β-catenin was more significantly reduced (Fig. 4B). In addition, the expression of EMT related proteins N-cadherin, Vimentin, and snail was significantly reduced, while the expression of E-cadherin was significantly increased (Fig. 4B). These results indicated that MPP7 was likely to affect the epithelial mesenchymal transition of epithelial ovarian cancer cells through Wnt/β-catenin signaling pathway, thereby promoting the progression of ovarian cancer.

In order to further explore the impact of MPP7 on polarity changes in ovarian cancer cells, we performed planar scratching on ovarian cancer cells and combined immunofluorescence co-localization to analyze the effect of MPP7 on the polarity of ovarian cancer cells through the localization changes of Golgi apparatus in ovarian cancer cells. As shown in the schematic diagram of Figure 4C, with the scratch side as the pointing coordinate, if the GM130 labeled Golgi apparatus was located within 120 ° range in front of the nucleus which was defined as "Cell directed migration".

After interfering with MPP7, the directional migration rate of cells was significantly reduced compared to the control group (Fig. 4C), and the difference was statistically significant (*P* < 0.01). The above results indicated that interfering with the expression of MPP7 could affect the polarity of ovarian cancer cells and inhibit their directed migration.

## Discussion

We analyzed the expression of MPP1-MPP7 in the TCGA/GTEx database and found that only the expression level of MPP7 was significantly increased in ovarian cancer samples. Multiple datasets in the GEO database further confirmed the abnormally high expression of MPP7 in ovarian cancer tissue. Therefore, we chose MPP7 as the target gene for our study in ovarian cancer.

MAGUK P55 scaffold protein 7 (MPP7) is a member of the Stardust family of membrane bound guanosine protein P55, which forms a ternary complex with Discs Large 1 (DLG1) and Lin7 to regulate cell connectivity 24. MPP7 plays an important role in establishing epithelial cell polarity by interacting with the polar protein DLG1 25. Some studies have shown that the change of MPP7 expression in pancreatic islets may affect cell polarity, damage glucose induced insulin secretion, and lead to diabetes 26. Other studies on tumors have shown that MPP7 can promote autophagy in pancreatic ductal adenocarcinoma cells by activating YAP1 22; MPP7 expression in breast cancer tissue is significantly higher than that in normal tissue, overexpression of MPP7 can promote the migration and invasion of breast cancer cells 23; Interference with MPP7 can inhibit the migration and invasion of esophageal cancer cells, and it can serve as a new biomarker for esophageal cancer 27; MPP7 is associated with tumor metabolism and immune infiltration in renal clear cell carcinoma and is a potential prognostic marker 28. In addition, the expression of MPP7 in metastatic tumors is significantly increased and positively correlated with tumor staging 23. And another large-scale proteomic analysis found that MPP7 may be a potential therapeutic target for serous ovarian cancer 29. However, how MPP7 affects the biological behavior of ovarian cancer cells and its specific molecular mechanisms have not yet been elucidated.

We found that patients with high expression of MPP7 had poorer prognosis through survival analysis in the Kaplan Meier plotter database. Then we detected the expression of MPP7 in benign ovarian epithelial cysts and epithelial ovarian cancer tissue with tissue chip immunohistochemistry, the results also showed that MPP7 was significantly overexpressed in epithelial ovarian cancer tissue, and its high expression was closely related to late clinical stage, high pathological grade, and lymph node metastasis, indicating that MPP7 might be an effective indicator for predicting poor prognosis in ovarian cancer patients.

We conducted a series of cell function experiments by interfering with MPP7 in ovarian cancer cells, and the results showed that interfering with MPP7 could inhibit the proliferation, migration, and invasion of ovarian cancer cells. Given the unique properties of MPP7, we further conducted cell scratch experiments and performed planar polarity immunofluorescence staining. We found that interfering with the expression of MPP7 could affect the polarity of ovarian cancer cells and inhibit their directed migration.

To further clarify the molecular mechanism by which MPP7 plays a role in ovarian cancer, we conducted GESA analysis using RNA sequencing data from the TCGA ovarian cancer database and found that MPP7 was significantly associated with Wnt/β-catenin pathway and EMT. The classic Wnt signaling pathway has been proven to be a key pathway in promoting the malignant phenotype of cancer, involved in regulating biological processes such as cell proliferation, differentiation, and apoptosis, and closely related to various cancers in humans, including ovarian cancer 30. The experimental results of this study indicated that MPP7 could promote the activation of Wnt10b, thereby promoting intracellular phosphorylation of β-catenin, thereby regulating the expression of the oncogene c-MYC downstream of the Wnt/β-catenin signaling pathway.

Research has shown that EMT is one of the key initiating events in the metastasis cascade reaction, which can endow tumor cells with the ability to migrate and invade 31. EMT is activated during tumor invasion 32. The loss of epithelial differentiation and acquisition of mesenchymal phenotype cause cancer cells to separate from the primary tumor mass and divide into the surrounding stroma 33. EMT induced transcriptional inhibitors such as ZEB1 can mediate the loss of E-cadherin expression, leading to impaired cell adhesion function, cell detachment, and nuclear translocation of β-catenin 34-36. More and more evidence suggests that E-cadherin and β-catenin interact with EMT induced transcriptional inhibitors in various ways to stabilize the invasive mesenchymal phenotype of epithelial tumor cells 37. In cancer, EMT enables epithelial phenotype cancer cells to acquire stromal features, characterized by decreased expression of E-cadherin and increased expression of Vimentin and N-cadherin 38. We detected the expression of EMT related proteins in ovarian cancer cells after interference with MPP7. We found that silencing MPP7 could inhibit the expression of Vimentin and N-cadherin, and promote the expression of E-cadherin, indicating that MPP7 could promote the EMT ability of ovarian cancer cells. The above results indicated that MPP7 might mediate EMT through the Wnt/β-catenin signaling pathway, thereby promoting the development of ovarian cancer, suggesting that MPP7 might become an effective potential target for the treatment of ovarian cancer.

In summary, MPP7 might promote the polarity change of ovarian cancer through EMT mediated by the Wnt/β-catenin signaling pathway, leading to the progression of ovarian cancer. MPP7 could have the potential to become a new prognostic biomarker and therapeutic target for ovarian cancer.

## Figures and Tables

**Table 1:** Relationship between the expression level of MPP7 and the clinical and pathological characteristics of ovarian cancer [n (%)].

		MPP7 Low	MPP7 High	Total	χ2	*P* value
Type	EOC	35 (36.5)	61 (63.5)	96	84.506	0.000
	Ovarian cyst	131 (92.3)	11 (7.7)	142		
	Total	166	72	238		
Grade	high-grade	10 (15.4)	55 (84.6)	65	38.588	0.000
	low-grade	25 (80.6)	6 (19.4)	31		
	Total	35	61	96		
Stage	I+II	32 (68.1)	15 (31.9)	47	39.758	0.000
	III+IV	3 (6.1)	46 (93.9)	49		
	Total	35	61	96		
lymph node	positive	1 (2.4)	40 (97.6)	41	35.751	0.000
	negative	34 (61.8)	21 (38.2)	55		
	Total	35	61	96		
Age	≤50	17 (47.2)	19 (52.8)	36	2.881	0.125
	>50	18 (30.0)	42 (70.0)	60		
	Total	35	61	96		
